# Supplementary material for: Structure-Dependent Toxicokinetics of Selected Pyrrolizidine Alkaloids In Vitro
Source: Int J Mol Sci. 2022 Aug 16;23(16):9214. doi: 10.3390/ijms23169214 (PMC9408898; doi:10.3390/ijms23169214)
Supplement: Supplementary file 1 [file ijms-23-09214-s001.zip › ijms-1825124-supplementary.pdf]

- Supplementary Materials-

Structure-Dependent Toxicokinetics of Selected Pyrrolizidine Alkaloids In Vitro

Julia Buchmueller [1], Florian Kaltner [2,3], Christoph Gottschalk [1,2], Maria Maares [1,4], Albert Braeuning [1] and Stefanie Hessel-Pras [1,*]

[1] German Federal Institute for Risk Assessment, Max-Dohrn-Str. 8-10, 10589 Berlin, Germany
[2] Chair of Food Safety, Veterinary Faculty, Ludwig-Maximilians-Universität München, Schoenleutnerstr. 8, 85764 Oberschleissheim, Germany
[3] Institute of Food Chemistry and Food Biotechnology, Justus Liebig University of Giessen, 35392 Giessen, Germany
[4] Institute of Food Chemistry and Toxicology, Technische Universität Berlin, Straße des 17. Juni 135, 10623 Berlin, Germany
* Correspondence: stefanie.hessel-pras@bfr.bund.de; Tel.: +49-30-18412-25203

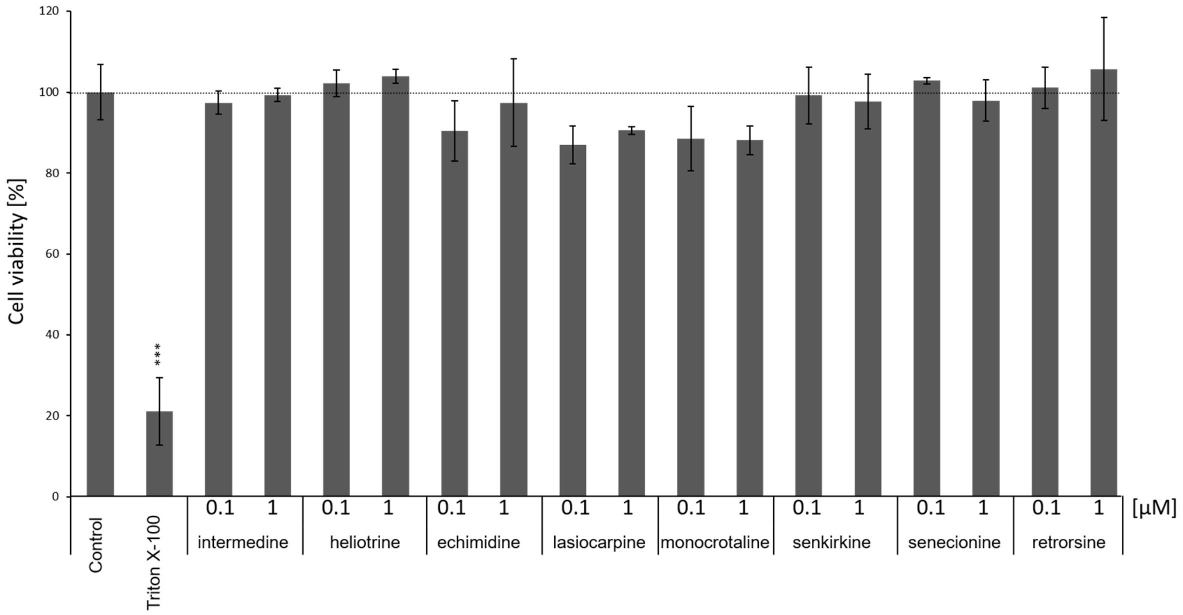

Figure S1. Cell viability of Caco-2 cells. 5000 cells per well were seeded and allowed to differentiate for 21 days. Next, they were incubated with 0.1 or 1 µM of the specific PAs for 24 h at 37 °C. Cell viability was detected by adding 20 µl WST solution to each well and incubate for 20 min at 37 . The absorption was detected with a TecanM200 Pro spectrometer at λ = 450 nm. Three individual experiments were performed. Triton X-100 was used as a positive control. Statistical significance was determined with One Way ANOVA followed by Dunnett´s posthoc test and indicated as *** p < 0.001.
